# ﻿Forest-dwelling crab-eating foxes, *Cerdocyon thous* (Carnivora, Canidae), in eastern Ecuador: new ecological and genetic records of an elusive Neotropical canid

**DOI:** 10.3897/zookeys.1266.162023

**Published:** 2026-01-08

**Authors:** Fernando Anaguano-Yancha, Ana Lucía Pilatasig, Laura Simba, Mauricio Herrera-Madrid, Galo Zapata-Ríos, Jorge Brito

**Affiliations:** 1 Wildlife Conservation Society, Programa Ecuador, Av. Mariana de Jesús O7-248 y La Pradera, Quito, Ecuador Wildlife Conservation Society, Programa Ecuador Quito Ecuador; 2 Pontificia Universidad Católica del Ecuador, Facultad de Ciencias Exactas y Naturales, Museo de Zoología-Sección de Mastozoología, Av. 12 de octubre 1076 y Roca, Quito, Ecuador Pontificia Universidad Católica del Ecuador Quito Ecuador; 3 Instituto Nacional de Biodiversidad, Quito, Ecuador Instituto Nacional de Biodiversidad Quito Ecuador

**Keywords:** Amazon rainforest, diet, distribution, mitochondrial DNA, parasitism, roadkill records, Yasuni National Park, wildlife conservation

## Abstract

The crab-eating fox, *Cerdocyon thous*, is the most broadly distributed native canid in South America, occupying a wide range of ecosystems from open savannas to dense tropical forests from Venezuela to Argentina. Its geographical range is expanding towards the Pacific coast of Central America by crossing the Darien rainforest. In Ecuador, its presence has been poorly documented and remains ecologically ambiguous. In this study, we confirm the presence of *C. thous* in north-eastern Ecuador based on two roadkill specimens collected in Sucumbíos Province and two independent photographic records from Yasuni National Park, which were obtained through systematic camera-trapping. Stomach-content analysis revealed a predominantly insectivorous and opportunistic diet which includes insects, arachnids, reptiles, and amphibians. In addition, an ectoparasite analysis found parasitism by the introduced cattle tick *Rhipicephalus microplus*, highlighting potential risks of pathogen transmission between domestic animals and wildlife. Mitochondrial cytochrome b sequences from form a well-supported distinct haplotype from other South American lineages, suggesting that forest-dwelling foxes in eastern Ecuador may represent a separate evolutionary unit with unique ecological adaptations to rainforest environments. These findings underscore the importance of continued monitoring of *C. thous* in the Amazon, particularly in the context of road expansion, habitat alteration, and increasing interactions with domestic animals such as domestic dogs. Our results have implications for taxonomic assessment, ecological understanding, and conservation planning of this widespread, yet understudied, Neotropical canid in Ecuador.

## ﻿Introduction

The crab-eating fox, *Cerdocyon thous* (Linnaeus, 1766), is the wild canid with the broadest distribution in South America (Maffei and Taber 2003). It ranges from northern Venezuela to Argentina (Lucherini 2015; Castelló 2018). Notably, recent records suggest that this species is expanding its range from the south-eastern Atlantic region of South America to the central Pacific coast of Central America by crossing the Darién forests (Tejera et al. 1999; Hody et al. 2019). Furthermore, its distribution extends from north-eastern to southern Argentina (Fariñas-Torres et al. 2024; Manzione et al. 2025). *Cerdocyon thous* is a highly adaptable canid, occurring in diverse ecosystems, including tropical and subtropical forests, montane regions, dry areas, plains, and savannas, from sea level to altitudes of 3690 m (Escobar-Lasso et al. 2016; Castelló 2018).

*Cerdocyon thous* exhibits an omnivorous and opportunistic feeding strategy, consuming a wide variety of food items including fruits, plants, molluscs, insects, fish, amphibians, reptiles, birds, and both small and medium-sized mammals (Bueno and Motta-Junior 2004; Gatti et al. 2006; Pedó et al. 2006; Canesini et al. 2008; Dutra-Vieira et al. 2024). This dietary flexibility allows it to exploit diverse habitats and seasonal food availability (Gatti et al. 2006; Pedó et al. 2006). However, this species is also known to prey on small domestic animals, such as poultry, which frequently leads to conflict with local communities. These interactions often result in negative perceptions and, in some cases, retaliatory killing or persecution as a means of conflict resolution (Kihn et al. 2022).

This species is known for its ecological plasticity and tolerance of anthropogenic disturbance (da Silva et al. 2020). It occupies a range of modified habitats, including grasslands, agricultural areas, and peri-urban and urban settings (Dias et al. 2014; Jiménez et al. 2017; da Silva et al. 2020). However, such tolerance also exposes it to significant threats, particularly road mortality, making *C. thous* one of the most frequently reported mammal species in wildlife–vehicle collisions across its geographical range (Vázquez Rodríguez et al. 2016; Bauni et al. 2017; Rojano Bolaño and Ávila Avilán 2021). In peri-urban areas, *C. thous* often interacts with domestic animals, in particular with free-ranging dogs, *Canis lupus familiaris* (Linnaeus, 1758), which compete for similar resources such as prey (Lacerda et al. 2009; Sánchez-Londoño 2014; Lessa et al. 2016) and territory (Jiménez et al. 2017). These interactions can alter the activity patterns of *C. thous* (Jiménez et al. 2016), increase disease exposure (Brandão et al. 2020; Acevedo and de Lima 2021; Dutra-Vieira et al. 2024), and facilitate the bidirectional transmission of pathogens (Nava et al. 2017; Ramos et al. 2020; Jiménez-Ruiz et al. 2024), particularly in peri-urban areas where anthropogenic ecosystem changes have intensified the spatial overlap between wildlife and domestic animals.

*Cerdocyon thous* exhibits considerable ecological variation across its range, potentially representing distinct evolutionary and ecotypic forms adapted to local environments (Martinez et al. 2013; Santos et al. 2024). Populations are known to be omnivorous and nocturnal in open areas, such as the Cerrado and Pampas biomes (de Matos Dias and Bocchiglieri 2016). Forest-dwelling individuals, especially those in the Amazon, may exhibit different foraging strategies, trophic roles, and habitat-use patterns (Castelló 2018; Dutra-Vieira et al. 2024). Populations that occupy mosaics composed of native and exotic vegetation tend to have a generalist diet, consuming a mix of animal prey, and both native and exotic fruit and other plant materials (Rocha et al. 2008), and animals may modify their activity patterns based on factors such as human density and available habitat (Santos et al. 2024). Such differences suggest that *C. thous* may exhibit localised adaptations despite taxonomic continuity (Dutra-Vieira et al. 2024), with important implications for understanding its ecological niche, ecological plasticity, and for informing conservation strategies across its broad geographic distribution.

Despite its broad range, *C. thous* remains a rare and poorly understood species in Ecuador. Historical records suggest its occurrence in the Andes and the coastal regions of the country (Tirira 2009; Lucherini 2015; Ramírez-Chaves and Pérez 2015). However, evidence of its presence in Ecuador is limited to a single specimen (Tirira 2009), leading to uncertainty and controversy over its historical and current occurrence in Ecuador (Ramírez-Chaves and Pérez 2015). Since the 1990s, however, there have been increasing reports of this species in the northern Amazon of Ecuador (Tirira 2017). Two main hypotheses have been proposed to explain these observations: that they represent sporadic occurrences, or that they are the result of a recent range expansion into the Amazonian lowlands (Tirira et al. 2018). The objective of this study was to confirm the presence of *C. thous* in the northern Ecuadorian Amazon, using verified roadkill specimens and camera trap detections, and to provide new information on this species’ diet, ectoparasites, and mitochondrial genetic variation. These data offer novel insights into the ecology, biogeography, and potential differentiation of *C. thous* populations in the tropical rainforests of eastern Ecuador.

## ﻿Methods

### ﻿Fieldwork with camera traps

**Figure 1.**
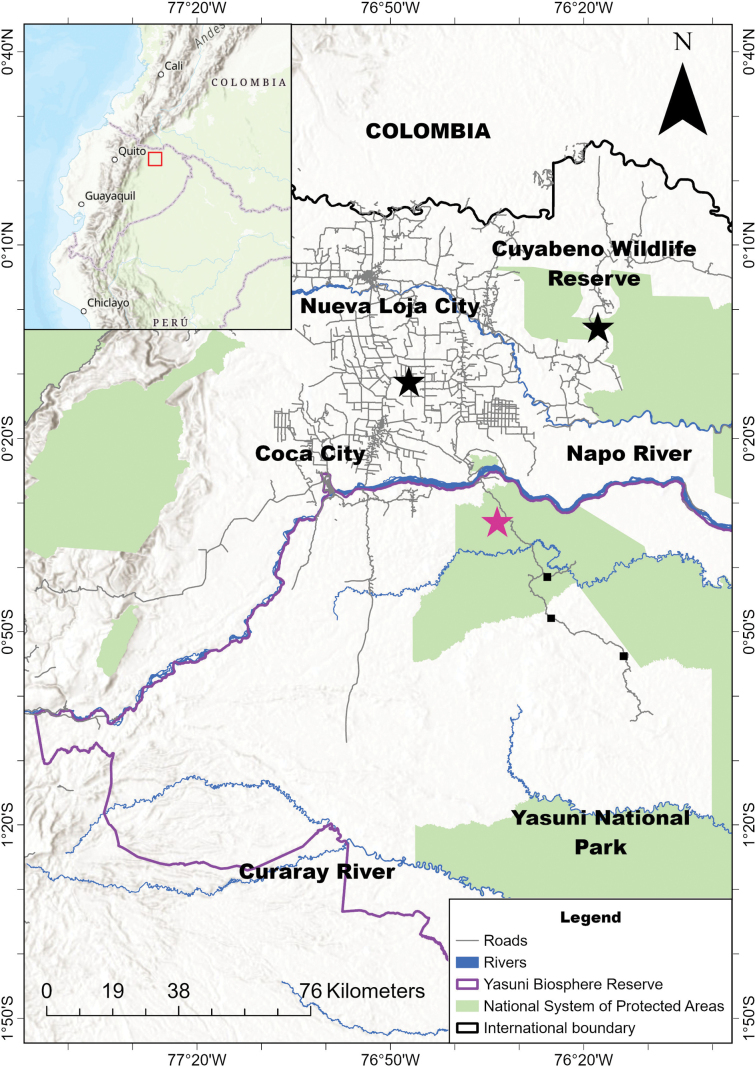
Record localities of *Cerdocyon thous* in the northern Amazon of Ecuador. Records of roadkill specimens (black stars); photographic records obtained in the photo-trapping campaigns (purple star), and direct observations previous (black squares) by Tirira et al. (2018).

Between 2015 and 2019, we conducted two camera-trapping campaigns (CFT2015 and CFT2018), in an area of approximately 920 km^2^ in the western section of Yasuni Biosphere Reserve and its buffer zone. For each campaign, we deployed 115 trail camera stations, across an altitudinal gradient from 173 to 728 m (Fig. 1). Each station was equipped with a Reconyx HC650 camera trap (Reconyx, Holmen, WI, USA), programmed to capture five photographs per trigger event, with a 60-s delay between events. Cameras were installed 30–50 cm above the ground and remained continuously active, 24 h per day, for 7–52 consecutive days between checking. We did not use any short-distance attractant to maximize capture success rates. Fieldwork was conducted under research permits issued by the Ministerio de Ambiente y Energía de Ecuador (MAE): MAE-DPAP-PIC-FAUNA-2015010, 028-2015-FAU-MAE-DPAO-PNY, AC-FAU-MAE-DPAP-2018-04, 002-2018-IC-PNY-DPAO/AVS).

### ﻿Roadkill specimens

In 2022, the Mammalogy Section of the Zoology Museum at the Pontificia Universidad Católica del Ecuador (QCAZ) received a *C. thous* roadkilled specimen from a secondary road in the community Los Laureles (00°02'45"S, 076°17'46"W; 260 m alt.), Sucumbíos Province. In 2024, the Instituto Nacional de Biodiversidad (MECN) received a second roadkilled specimen from the area of Cóndor Mirador (00°11'08.6"S, 076°47'05.5"W; 280 m alt.), also in the province of Sucumbíos (Fig. 1). Before preparation, we carefully examined each specimen for ectoparasites, which were manually collected using forceps and preserved in 75% ethanol. Specimens were subsequently dissected and their stomachs removed following the protocol described by Benavides Melo et al. (2021) and stored at −10 °C until further analysis. We prepared both specimens as study skins, with associated skulls, skeletons, bacula, and soft tissues preserved and deposited them in the mammal collections of QCAZ 19152 (PV648362), and MECN 8300 (PV648361).

### ﻿Identification and analysis of information

Photographic records and roadkill specimens were identified based on morphological characteristics described in Tirira (2017), Castelló (2018), and Webb and Blincow (2024). We calculated the photographic capture rate as the number of independent photographic events per 100 trap-nights. We considered a photographic event independent if at least 1 h had elapsed between consecutive detections of the same species at the same camera station (Rovero et al. 2014).

### ﻿Stomach contents

To analyse the stomach contents, we thawed the samples and made a longitudinal incision from the pylorus to the cardia. Contents were emptied into a plastic tray and examined under a Relife RL-M3T stereomicroscope. We classified the prey items into five broad categories: reptiles, amphibians, invertebrates, plant material, and unidentifiable remains. Vertebrate remains were identified to the lowest possible taxonomic level (typically class or order, and in some case to species) using the BIOWEB repository (https://bioweb.bio/). Invertebrate identification was conducted using standard references (e.g. Triplehorn and Johnson 2005; Chapman 2013; Gullan and Cranston 2014) and by consulting taxonomic specialists. We estimated the relative proportion of each food item using the gravimetric method (Hyslop 1980).

### ﻿Ectoparasites

We identified ectoparasites to the species level following Nava et al. (2017). We photographed the specimens using a Relife RL-M3T stereomicroscope equipped with a Sunshine 48000W camera (48 MP). All ectoparasites are deposited in the QCAZ collection.

### ﻿DNA extraction and analysis

To verify the taxonomic identity of the roadkill specimens, we extracted DNA from liver tissue using the GeneJET Genomic DNA Purification Kit (K0722), following the manufacturer’s instructions. We amplified the complete mitochondrial cytochrome *b* (Cyt-*b*) gene by PCR using the primers MTCB-forward (5′-CCHCCATAAATAGGNGAAGG-3′) and MTCB-reverse (5′-WAGAAYTTCAGCTTTGGG-3′) (Naidu et al. 2011) and the GoTaq® Green Master Mix 2X kit. Each reaction was prepared in a final volume of 10 µL, with the following composition (in µL): 2.1 of GoTaq, 0.4 of each primer, 0.1 of BSA (bovine serum albumin), and 1 of DNA extract. PCR conditions included an initial denaturation phase at 95 °C for 6 min, followed by 36 amplification cycles. Each cycle included denaturation at 95 °C for 45 s, annealing at 52 °C for 1 min, extension at 72 °C for 2 min, with a final extension step at 72 °C for 10 min. We sequenced the Cyt-*b* gene using a MinION mk1c equipped with Flongle Flow Cells R v. 10.4.1 and the Rapid Barcoding Kit 96 (SQK-RBK114.96), following standard protocols. Dorado v. 4.3.0 was used for base calling. All molecular analyses were conducted at the Nucleic Acid Sequencing Laboratory at INABIO, Quito, Ecuador. The genetic study was conducted under authorization of access to genetic resources no. MAATE-DBI-CM-2023-0334, issued by the Ministerio de Ambiente y Energía de Ecuador.

### ﻿Sequence analysis

The resulting fastq files were quality-filtered using a minimum Q score of 9. Consensus sequences were generated using NGSpeciesID v. 0.3.0 (Sahlin et al. 2021). For subsequent phylogenetic analyses, sequence alignments were performed in MAFFT as implemented in Mesquite, and sites containing gaps or missing data were retained and treated as missing characters rather than being removed (i.e. no complete deletion was applied). We aligned our sequences with eight published *C. thous* Cyt-*b* sequences from GenBank (four from Brazil, Carnieli et al. 2016; and three from Argentina, Chemisquy et al. 2019) using the MAFFT algorithm in Mesquite v. 3.81 (Maddison and Maddison 2023). A maximum-likelihood phylogenetic tree (ML) was constructed in MEGA v. 12 (Kumar et al. 2024) using the Hasegawa-Kishino-Yano substitution model (Hasegawa et al. 1985). The best tree was manually rooted using an outgroup branch in MEGA v. 12 (Kumar et al. 2024). Pairwise genetic distances were also calculated using the same program. The *C. thous* Cyt-*b* sequences generated in this study have been deposited in GenBank under accession numbers PV648361 (MECN 8300) and PV648362 (QCAZ 19152).

## ﻿Results

### ﻿Camera trap records

**Figure 2.**
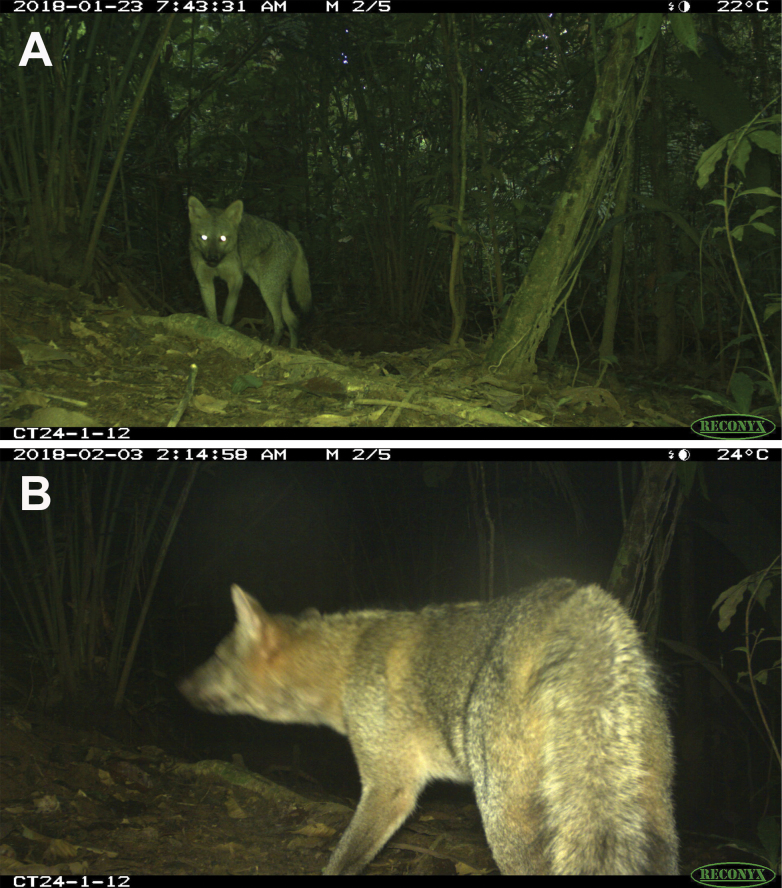
Photographic records of *Cerdocyon thous* obtained during the camera-trapping campaign conducted in 2018 in Yasuni Biosphere Reserve. **A.** Front view; **B.** Lateral view.

During the CFT2018 campaign, with a sampling effort of 4383 trap-nights, we recorded two independent photographic events of *Cerdocyon thous* (Fig. 2). Both events were captured at a camera trap station installed in the Kichwa community of Pompeya, Yasuni National Park (00°32'52"S, 076°33'23"W; 240 m alt.). The detections occurred on 23 January 2018, at 07:43, and on 3 February 2018, at 02:14 (Fig. 2A, B). The estimated photographic capture rate for *C. thous* was low, at 0.045 (± 0.06 SE) independent events per 100 trap-nights. In comparison, the capture rate for the short-eared fox, *Atelocynus microtis* (Sclater, 1883), was higher at 0.19 (± 0.11 SE), while the rate for domestic dogs was 0.42 (± 0.16 SE). The rate for the bush dog, *Speothos venaticus* (Lund, 1839), was lower at 0.02 (± 0.017 SE).

### ﻿Morphological characteristics

**Figure 3.**
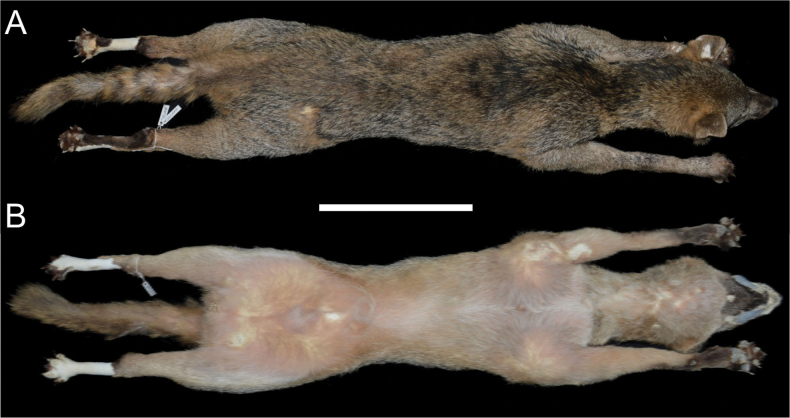
Skin of *Cerdocyon thous* (QCAZ 19152) from Los Laureles, Sucumbíos Province, Ecuador. **A.** Dorsal view; **B.** Ventral view. Scale bar: 300 mm.

The fur of *C. thous* specimens (QCAZ 19152 and MECN 8300) is coarse and moderately long, with a greyish-brown dorsum in the juvenile specimen and a yellowish-brown dorsum in the adult (Fig. 3). In both individuals, black hair was intermixed throughout the dorsal pelage. A blackish mid-dorsal stripe was visible, extending from the nape to the base of the tail in the juvenile (MECN 8300), and to the mid-dorsum in the adult (QCAZ 19152). The ventral areas are whitish, with the neck and undersides appearing greyish. Both specimens exhibited pale ear tips, a black occiput, and a black mandible. The tail was moderately bushy, with dark colouration along the dorsal surface in the juvenile, while the adult showed orange stripes.

**Figure 4.**
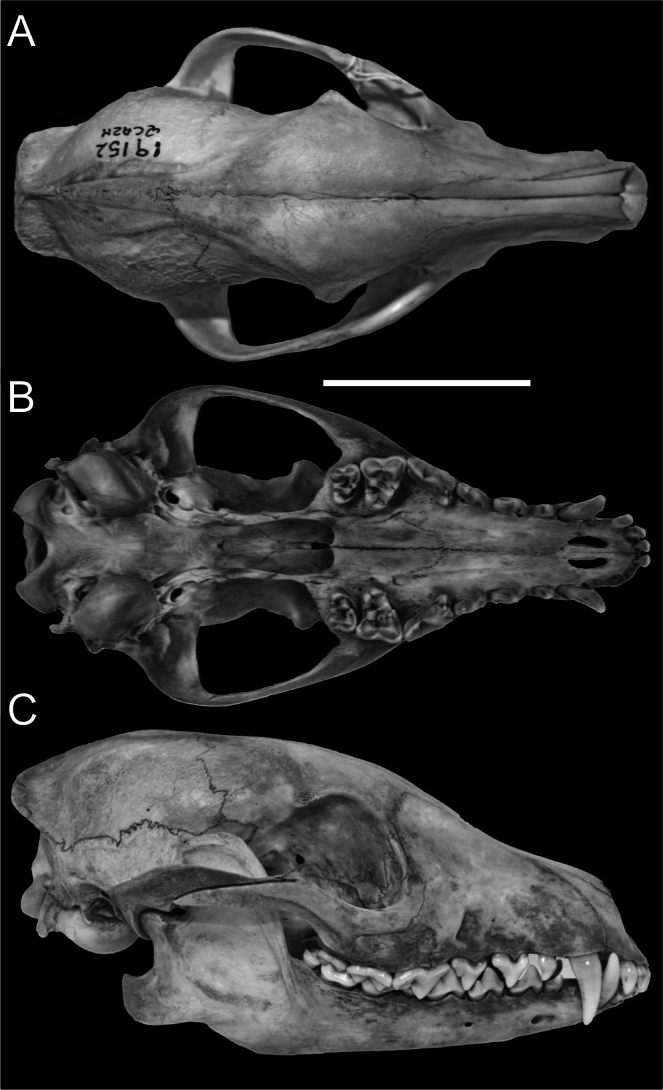
Skull of *Cerdocyon thous* (QCAZ 19152) from Los Laureles, Sucumbíos Province, Ecuador. **A.** Dorsal view; **B.** Ventral view; **C.** Lateral view. Scale bar: 50 mm.

Skull morphology (Fig. 4) was characterized by a relatively short snout, a flattened and lyriform sagittal region with striated outer-posterior margins, a convex forehead, well-developed frontal sinuses, and a palatine bone that is shorter than the dental row. The vertebral column comprised 19 thoracicolumbar vertebrae, four fused sacral vertebrae, and 18 caudal vertebrae. There were 12 ribs. Body and cranial measurements are detailed in Table 1.

**Table 1.** Body and cranial measurements of *Cerdocyon thous* specimens. Cranial measurements follow the proposal of Neto et al. (2020).

Measurements (in mm)	QCAZ 19152 (adult male)	MECN 8300 (juvenile male)
Head–body length	701	450
Tail length	320	260
Ear length	72	65
Leg length	120	135
Weight (kg)	8	3
Cranial height	36	40
Length of the skull	145.3	115
Length of the neurocranium	84.1	68
Length of the viscerocranium	64.6	56
Width of the neurocranium	48.2	45
Zygomatic width	75.3	63
Length of the base of the skull	131.7	105
Condylobasal length	138.7	113
Length of the nose	67.2	57
Length of the nasal bones	49.3	40
Length of the mandible	114.1	84
Palatal length	64.6	55
Width between the jugular processes	49.9	36
Width between the occipital condyles	31	26
Width of the foramen magnum	16.7	15
Height of the occipital triangle	32	30
Height of the foramen magnum	11.3	10.5

### ﻿Stomach contents

**Figure 5.**
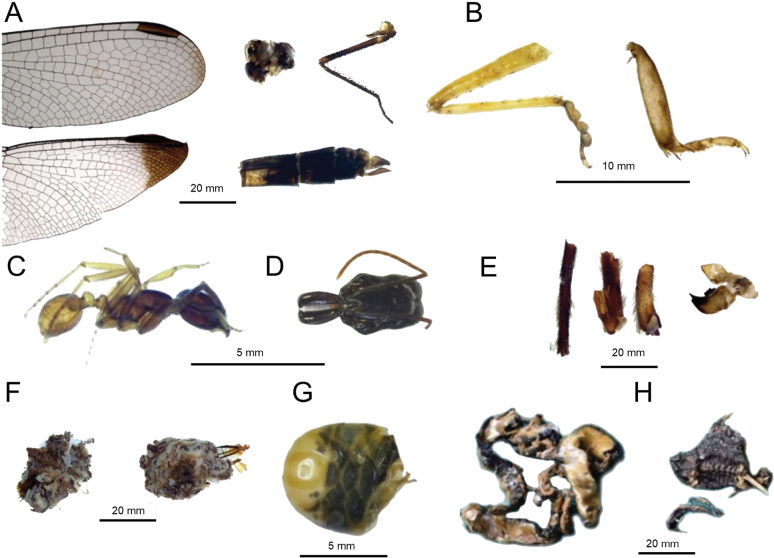
Main prey consumed by *Cerdocyon thous* (QCAZ 19152) from Los Laureles, Sucumbíos Province, Ecuador. **A.**Odonata (wings, head, abdomen and leg); **B.** Hind leg of Orthoptera, and Blattodea leg; **C.** Habitus of *Labidus* sp. (Hymenoptera, Formicidae); **D.** Head of *Odontomachus* sp. (Hymenoptera, Formicidae); **E.** Parts of legs and mouthparts of Arachnidae; **F.** Digested material; **G.** Head and body of *Amerotyphlops reticulatus* (Squamata, Typhlopidae); **H.***Rhinella marina* (Anura, Bufonidae).

Analysis of the stomach contents from specimen QCAZ 19152 yielded a total wet mass of 39.43 g, from which 468 identifiable items were recovered (Fig. 5A–H). Insects represented most of the consumed biomass (21.24 g; 54%). Notable insect taxa included two families of Odonata (Gomphidae and Libellulidae), as well as members of the family Formicidae (Hymenoptera), specifically the genera *Labidus* and *Odontomachus*. Additional invertebrate prey included representatives of the orders Blattodea and Orthoptera, along with various arachnids. Vertebrate prey (8.67 g; 22%) included one individual each of *Amerotyphlops reticulatus* (Linnaeus, 1758) (Squamata, Serpentes, Typhlopidae) and *Rhinella marina* (Linnaeus, 1758) (Anura, Bufonidae). Plant material contributed 7% (2.72 g) of the stomach content. Due to advanced digestion, 17% (6.8 g) of the content could not be identified to any taxonomic group.

### ﻿Ectoparasites

**Figure 6.**
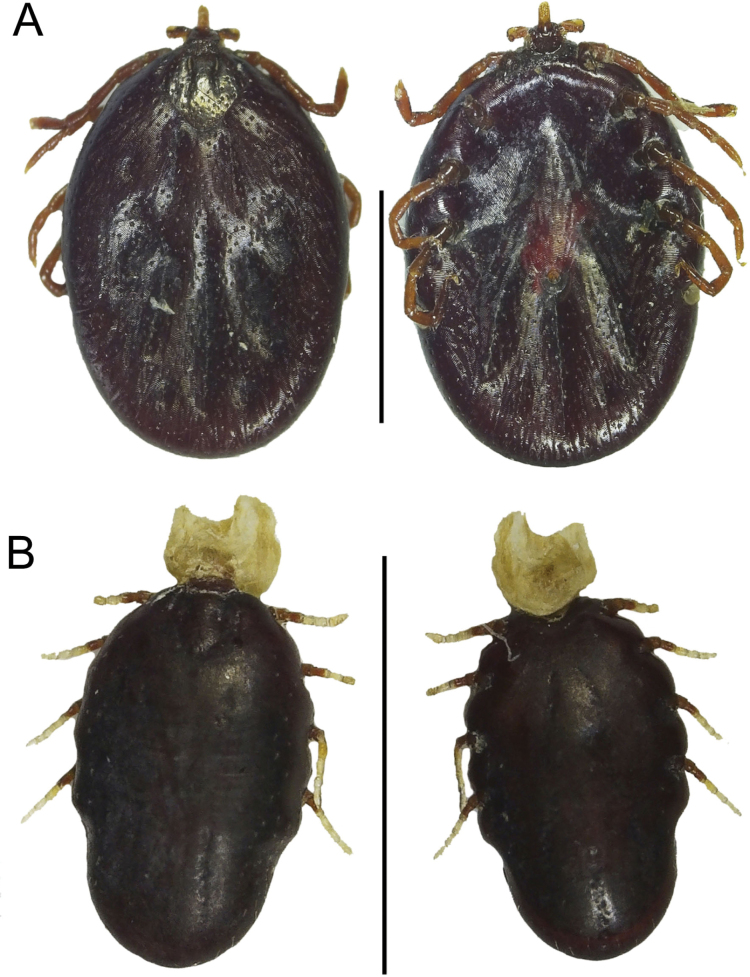
*Rhipicephalus microplus* (Ixodida, Ixodidae) collected on *Cerdocyon thous* (QCAZ 19152) from Los Laureles, Sucumbíos Province, Ecuador. **A.** Dorsal and ventral view of an adult female; **B.** Dorsal and ventral view of a nymph. Scale bar: 10 mm.

Six ticks were recovered from the neck region of specimen QCAZ 19152. These included two adult females and four nymphs of *Rhipicephalus microplus* Canestrini, 1888 (Ixodida, Ixodidae) (Fig. 6). This tick is characterized by a dorsally hexagonal basis capituli and having palps shorter than the hypostome. The hypostome is short, blunt, and with a dental formula of 4/4. The genital aperture is located between coxa II and III. This finding constitutes the first documented case of *R. microplus* parasitising *C. thous* in Ecuador.

### ﻿Molecular analysis

**Figure 7.**
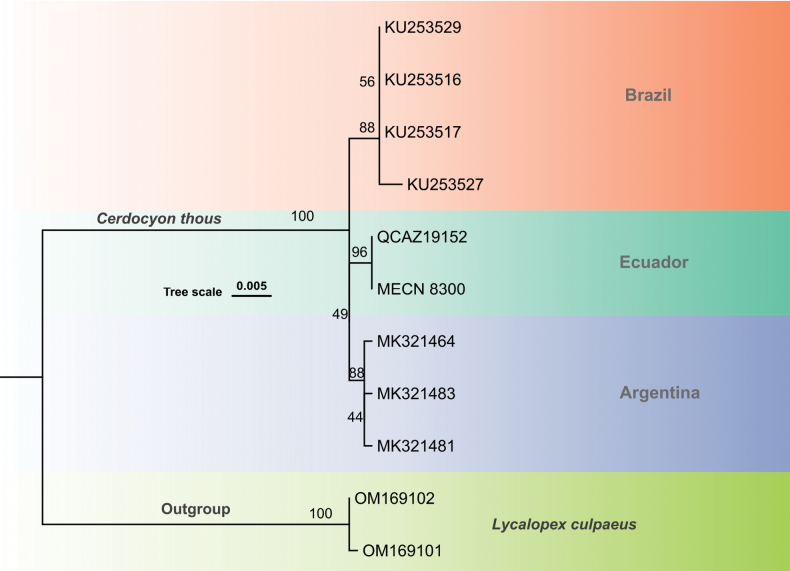
Maximum-likelihood phylogenetic tree inferred from the Cyt-*b* analyses of *Cerdocyon thous*. The tree includes the samples MECN 8300 and QCAZ 191552, from province of Sucumbíos, Ecuador. Branches show bootstrap support percentages.

Mitochondrial Cyt-*b* sequences (1140 bp) were successfully obtained from both MECN 8300 and QCAZ 19152. For the phylogenetic analyses, we used a 1024-bp fragment of the Cyt-*b* gene, which was compared with homologous sequences available in GenBank. Pairwise genetic distance analysis showed that the Ecuadorian specimens exhibited a divergence of 0.58% from individuals in Argentina and 0.68 from those in Brazil. The maximum-likelihood phylogenetic tree (Fig. 7) revealed that the Ecuadorian individuals form a well-supported haplotype, suggesting a distinct lineage potentially shaped by geographic or ecological isolation; however, inclusion of Colombian samples will be necessary to assess this hypothesis.

## ﻿Discussion

The specimens of *Cerdocyon thous* documented in this study represent some of the very few verified museum records of this species in Ecuador, reconfirming its presence in the country’s northern Amazon region. Although Tirira et al. (2018) hypothesized that *C. thous* may be expanding into Ecuador via recent colonization events, the evidence presented here (including low but confirmed photographic detections, roadkill records, and genetic distinctiveness) suggests that *C. thous* is present in intervening forest landscapes and areas of anthropogenic disturbance in the northern Amazon of Ecuador. Additional records are needed to determine whether these records represent a recent expansion or a long-established cryptic population. Three additional roadkill records from the northern Amazon on the citizen-science platform iNaturalist (https://ecuador.inaturalist.org/) suggest that *C. thous* is likely using road corridors to move through or into disturbed areas (Auz-Cerón et al. 2023; Santos et al. 2024). However, *C. thous* remains absent from national-scale roadkill datasets (Medrano-Vizcaíno et al. 2023), possibly due to low natural abundance or misidentification; *C. thous* is sympatric with two other native canid species in the Ecuadorian Amazon (Tirira et al. 2018; this study). Its absence from roadkill datasets is in contrast with high rates of roadkill reported for other areas of the Amazon region (Vázquez Rodríguez et al. 2016; Bauni et al. 2017; Rojano Bolaño and Ávila Avilán 2021).

Despite intensive camera-trap surveys across disturbance gradient in the northern Ecuadorian Amazon (Mena et al. 2020; Auz-Cerón et al. 2023; Jácome-Negrete et al. 2023), *C. thous* has rarely been recorded, implying either low abundance in intact forests or a strong preference for anthropogenically modified habitats. The low photographic capture rate in the western section of Yasuni Biosphere Reserve (this study) further supports this pattern, especially when compared to other canid species such as *A. microtis* and *S. venaticus*, which are recorded more frequently; this low capture rate is in contrast with assessments made in other areas of the species’ distribution (Jiménez-Alvarado et al. 2017; Caballero-Gini et al. 2020; Fonseca-Prada et al. 2022. Similarly, Fariñas-Torres et al. (2024) also reported low photographic rates in the southern portion of the species’ range, likely related to its occurrence near the edge of its known distribution. This scarcity may reflect either true rarity or avoidance behaviour (Smith et al. 2023; Crisfield et al. 2024). As a generalist species often associated with open or disturbed habitats, *C. thous* may be less suited to dense, undisturbed rainforest conditions (Maffei and Taber 2003; Jiménez et al. 2017; Castelló 2018).

Interspecific competition may also influence the occurrence of *C. thous*. Although direct evidence from Amazonian forests is lacking, studies in other regions (Bubadué et al. 2016) have shown that *C. thous* can compete with other canid species when sympatric, which could partly explain its tendency to use more degraded zones or ecotones rather than intact habitats (Maffei and Taber 2003; Jiménez et al. 2017; Castelló 2018). In these environments, it interacts with domestic mammals, such as domestic dogs, which are potential competitors for territory and food resources (Lacerda et al. 2009; Sánchez-Londoño 2014; Lessa et al. 2016; Jiménez et al. 2017) and are also vectors of pathogens that affect wild carnivores (Guzmán et al. 2024). In the western section of the Yasuni Biosphere Reserve, the photographic capture rate of domestic dogs increased in CFT2018 (0.42 ± 0.16 SE) compared to CFT2015 (0.05 ± 0.012 SE), indicating growing interactions between free-ranging dogs and native wildlife. This trend underscores the urgent need to implement measures that minimize dog–wildlife interactions, particularly in biodiversity-rich ecosystems such as Yasuni National Park.

The diet of *C. thous* has been the subject of several studies, especially in the Atlantic Forests and the Brazilian Pampa (Bueno and Motta-Junior 2004; Canesini et al. 2008; Dutra-Vieira et al. 2024). However, there are few reports on its diet in the Amazon rainforest (Dutra-Vieira et al. 2024). For the Ecuadorian Amazon, no data related to the diet of this canid have been documented (Vallejo 2022). The stomach-content analysis of the roadkill specimen from Dureno revealed a predominantly insectivorous diet, with insects and arachnids comprising nearly 40% of identifiable items, similar to that observed in other areas of the Brazilian Amazon (Dutra-Vieira et al. 2024). Vertebrate prey included one amphibian (*Rhinella marina*) and one fossorial snake (*Amerotyphlops reticulatus*). Although *C. thous* is known to consume snakes (Castellari Gonzalez et al. 2016; Dutra-Vieira et al. 2024) the presence of *A. reticulatus* in its diet represents a novel prey record for the species. These patterns are consistent with findings from forested areas in the Brazilian Amazon, where *C. thous* diet tends to include more invertebrates and small vertebrates compared to populations in more open environments (Dutra-Vieira et al. 2024). In contrast, studies from the Brazilian Cerrado, Atlantic Forest, and Pampas show diets richer in fruits, plant matter, and small to medium-sized mammals (Bueno and Motta-Junior 2004; Gatti et al. 2006; Pedó et al. 2006; Canesini et al. 2008). This opportunistic behaviour may reflect ecological adaptation to resource availability and lower visibility in dense forest environments, where large prey is harder to detect and capture. Such dietary plasticity reinforces the ecological generalist nature of *C. thous* but also highlights potential regional adaptations among forest-dwelling populations that warrant further investigation.

Across its geographical range, *C. thous* has been reported as a host for approximately 21 species of ticks. Most reports indicate a high prevalence of infestation by ticks of the genus *Amblyomma* (Ixodida, Ixodidae; Appendix 1), which commonly parasitise both wild and domestic animals during their adult stage (Nava et al. 2017). The presence of *R. microplus*, a species of tick introduced to the Neotropics from Europe via domestic cattle, represents the first such case of this parasite on *C. thous* in Ecuador. This ectoparasite has been previously recorded parasitising *C. thous* in Brazil and Venezuela (Jones et al. 1972; Rodrigues and Daemon 2004; Martins et al. 2014; Ramos et al. 2020) and is known to be a competent vector of various haemoparasites (Cruz-González et al. 2024). Its detection in a wild canid from a region undergoing increasing deforestation and cattle expansion raises concerns about spillover risks from domestic to wild species. Forest degradation may facilitate tick–host transmission cycles by increasing contact among wildlife, dogs, and livestock (Brandão et al. 2020; Cruz-González et al. 2024; Guzmán et al. 2024). The role of *C. thous* as a potential reservoir or host for pathogens in such altered landscapes merits focused study, especially in proximity to human settlements.

The mitochondrial Cyt-*b* analysis revealed that the Ecuadorian *C. thous* specimens form a distinct haplotype, with low but consistent genetic divergence from populations elsewhere. Phylogenetic analysis and genetic distance data suggest some geographic differentiation among South American populations of *C. thous*, although the limited sample size and reliance on mitochondrial markers constrain the strength of this inference. The Ecuadorian individuals form a well-supported haplotype; however, the possibility that they are related to populations from poorly sampled regions, such as trans-Andean Colombia, cannot be excluded. Brazilian specimens form a monophyletic group, while Argentine populations cluster closely in the phylogenetic tree; notably, the smallest observed genetic distance (0.58%) is between individuals from Argentina and Ecuador. This pattern contradicts the relative placement of these populations in the phylogenetic tree and suggests a more complex evolutionary history, potentially involving recent introgression or secondary contact, although additional data would be required to evaluate these possibilities. Currently, five subspecies (Cabrera 1931, 1958; Berta 1982; Bisbal 1988) of *C. thous* are recognized: *C. t. thous* (the Guianas, eastern Amazonia, and northern Brazil), *C. t. aquilus* (the savannas and forests of Colombia and Venezuela), *C. t. azarae* (north-eastern and central Brazil), *C. t. entrerianus* (southern Brazil, northern Argentina, Paraguay, western Bolivia, and Uruguay), and *C. t. germanus* (central Colombia); however, these require taxonomic evaluation to test their validity.

Representative genetic sequences are lacking for several subspecies and for many parts of the species’ geographical range, limiting our ability to confidently assign the observed lineages to any specific subspecies. However, our findings suggest some level of geographic structuring and, potentially, the emergence of a unique forest-associated evolutionary lineage. Although current data are insufficient to assign these individuals to a specific subspecies, their genetic differentiation, combined with ecological and geographical isolation, points to the possibility that the Ecuadorian population represents an evolutionarily significant unit (ESU). Additional sampling and integrative analyses, including morphological, genetic, and ecological data, are essential to test whether the Ecuadorian population corresponds to a previously recognised subspecies, represents a distinct geographic variant, or constitutes an undescribed evolutionary unit.

## ﻿Conclusions

This study presents the first verifiable ecological and genetic documentation of *Cerdocyon thous* in the Ecuadorian Amazon, confirming its presence through museum specimens, camera trap detections, and molecular data. Our findings challenge the prevailing notion of the species as a recent colonizer in the Ecuadorian Amazon, instead suggesting the existence of a cryptic, potentially established population that persists in disturbed forest mosaics shaped by anthropogenic activity.

The low capture rates in intact rainforest environments, contrasted with roadkill and photographic records from altered landscapes, indicate that *C. thous* may be functionally excluded from undisturbed habitats by ecological filters such as dense vegetation structure with limited prey visibility, and interspecific competition with forest-adapted canids. Dietary analysis revealed a predominantly insectivorous and opportunistic feeding strategy that differs from the frugivorous diets reported for populations in open biomes, underscoring the species’ remarkable ecological plasticity and potential local adaptations to Amazonian rainforest environments. The detection of the invasive cattle tick (*R. microplus*) on a wild *C. thous* individual signals not only increased contact with livestock and domestic animals, but also the emerging risk of pathogen spillover in landscapes undergoing rapid deforestation and agricultural expansion. Mitochondrial sequence data place the Ecuadorian individuals in a distinct, well-supported haplotype, contributing new evidence of geographical genetic structuring across the range of *C. thous* and hinting at the presence of a potentially unique evolutionary lineage in the north-western Amazon. While our data are insufficient to delimit a new subspecies or evolutionary significant unit formally, the combination of ecological, morphological, and genetic distinctiveness calls for further integrative taxonomic assessments.

Collectively, these results highlight the urgent need to reassess the conservation status of *C. thous* populations in Amazonian Ecuador. Future research should prioritize broader sampling across environmental gradients, the ecological role of *C. thous* in disturbed forests and its interactions with domestic species. Given its apparent association with anthropogenic habitats, *C. thous* may serve as a sentinel species for ecosystem change, zoonotic risk, and the resilience of medium-sized carnivores in fragmented Neotropical landscapes. Safeguarding its future requires not only taxonomic clarity but also the development of landscape-level conservation strategies that address habitat degradation, road expansion, and domestic dog-mediated disease transmission in one of the most biodiverse regions of the planet.

## ﻿Additional information

### Use of AI

No use of AI was reported.

### Author ORCIDs

Fernando Anaguano-Yancha https://orcid.org/0000-0001-5846-2230

Ana Lucía Pilatasig https://orcid.org/0000-0002-6956-570X

Laura Simba https://orcid.org/0009-0004-2442-7612

Mauricio Herrera-Madrid https://orcid.org/0000-0001-8060-2252

Galo Zapata-Ríos https://orcid.org/0000-0003-3508-5078

Jorge Brito https://orcid.org/0000-0002-3410-6669

### Data availability

All of the data that support the findings of this study are available in the main text.

## ﻿Appendix 1

**Table A1.** Cases of parasitism of ticks of the family Ixodidae reported on *Cerdocyon thous*.

Tick species	Phase			Country	Reference
	Larva	Nymph	Adult		
*Amblyomma aureolatum*	–	X	X	Brasil, Uruguay	Aragão and da Fonseca (1961); Rodrigues and Daemon (2004); Labruna et al. (2005); Martins et al. (2014); Nava et al. (2017); Beltrame et al. (2021)
*Amblyomma auricularium*	–	–	X	Venezuela	Jones et al. 1972; Guglielmone et al. (2003); Nava et al. (2017)
*Amblyomma brasiliense*	–	X	–	Argentina, Brasil	Labruna et al. (2005); Lamattina et al. (2014)
*Amblyomma cajennense*	X	X	X	Brasil, Venezuela	Jones et al. 1972; Labruna et al. (2005)
*Amblyomma coelebs*	–	X	–	Argentina	Lamattina et al. (2014)
*Amblyomma dubitatum*	–	X	X	Brasil	Labruna et al. (2005); Ramos et al. (2020)
*Amblyomma fuscum*	–	–	X	Brasil	Labruna et al. (2005)
*Amblyomma longirostre*	–	–	X	Brasil	Labruna et al. (2005); Nava et al. (2017)
*Amblyomma maculatum*	–	–	–	Venezuela	Jones et al. 1972
*Amblyomma neumanni*	–	–	X	Argentina	Guglielmone and Nava (2006), Nava et al. (2017)
*Amblyomma oblongoguttatum*	–	–	X	Panamá	Bermúdez et al. (2018)
*Amblyomma ovale*	–	X	X	Argentina, Brasil, Venezuela, Panamá	Aragão and da Fonseca (1961); Jones et al. (1972); Rodrigues and Daemon (2004); Labruna et al. (2005); Lamattina et al. (2014); Santos et al. (2016); Bermúdez et al. (2018); Ramos et al. (2020)
*Amblyomma parvum*	–	–	X	Brasil	Labruna et al. (2005)
*Amblyomma sculptum*	X	X	–	Brasil	Ramos et al. (2020); Beltrame et al. (2021)
*Amblyomma tigrinum*	X	–	X	Argentina, Brasil, Uruguay, Venezuela	Jones et al. 1972; Guglielmone et al. (2000), Labruna et al. (2005); Martins et al. (2014); Ramos et al. (2020)
*Amblyomma triste*	–	X	X	Brasil	Labruna et al. (2005); Nava et al. (2017)
*Amblyomma* sp	X	X	–	Brasil	Labruna et al. (2005); Ramos et al. (2020)
*Rhipicephalus microplus*	X	X	X	Brasil, Venezuela, Ecuador	Jones et al. 1972; Rodrigues and Daemon (2004); Nava et al. (2017); Ramos et al. (2020); This study
*Rhipicephalus sanguineus*	–	X	X	Brasil, Colombia, Panamá	Torres-Mejía (2006); Santos et al. (2016); Nava et al. (2017), Bermúdez et al. (2018)
*Dermacentor nitens*	–	–	X	Brasil, Venezuela	Jones et al. 1972; Labruna et al. (2005); Nava et al. (2017); Ramos et al. (2020)
*Haemaphysalis juxtakochi*	–	X	X	Panamá	Nava et al. (2017); Bermúdez et al. (2018)

**References**

Aragão H, da Fonseca F (1961) Notas de ixodologia: IX. O complexo ovale do gênero *Amblyomma*. Memórias do Instituto Oswaldo Cruz 59: 131–148. https://doi.org/10.1590/S0074-02761961000200002

Beltrame LB, Bernardi LF, Martins TF, Labruna MB, Favoretto SM, Guimarães AA (2021) Ticks (Acari: Ixodidae) in wild animals treated at the Federal University of Lavras, Minas Gerais State, Brazil. Entomological Communications 3: ec03036. https://doi.org/10.37486/2675-1305.ec03036

Bermúdez S, Apanaskevich D, Domínguez L (2018) Garrapatas Ixodidae de Panamá. Secretaría Nacional de Ciencia, Tecnología e Innovación, Instituto Conmemorativo Gorgas de Estudios de la Salud, Grupo de Estudios con Ectoparásitos, 129 pp.

Guglielmone AA, Mangold A, Luciani CE, Viñabal AE (2000) *Amblyomma tigrinum* (Acari: Ixodidae) in relation to phytogeography of central-northern Argentina with notes on hosts and seasonal distribution. Experimental y Applied Acarology 24(12): 983–989. https://doi.org/10.1023/A:1010775528628

Guglielmone AA, Estrada-Peña A, Luciani CA, Mangold AJ, Keirans JE (2003) Hosts and distribution of *Amblyomma auricularium* (Conil 1878) and *Amblyomma pseudoconcolor* Aragão, 1908 (Acari: Ixodidae). Experimental y Applied Acarology 29(1–2): 131–139. https://doi.org/10.1023/A:1024251020035

Guglielmone AA, Nava S (2006) Las garrapatas argentinas del género *Amblyomma* (Acari: Ixodidae): distribución y hospedadores. Revista de Investigaciones Agropecuarias 35(3): 133–153.

Labruna MB, Jorge RSP, Sana DA, Jácomo ATA, Kashivakura CK, Furtado MM, Ferro C, Perez SA, Silveira L, Santos Jr TS, Marques SR, Morato RG, Nava A, Adania CH, Teixeira RHF, Gomes AAB, Conforti VA, Azevedo FCC, Prada CS, Silva JCR, Batista AF, Marvulo MFV, Morato RLG, Alho CJR, Pinter A, Ferreira PM, Ferreira F, Barros-Battesti DM (2005) Ticks (Acari: Ixodida) on wild carnivores in Brazil. Experimental y Applied Acarology 36(1–2): 149–163. https://doi.org/10.1007/s10493-005-2563-1

Lamattina D, Tarragona EL, Costa SA, Guglielmone AA, Nava S (2014) Ticks (Acari: Ixodidae) of northern Misiones Province, Argentina. Systematic and Applied Acarology 19(4): 393. https://doi.org/10.11158/saa.19.4.2

Santos EM, Cunha RC, Farias MPO, Fonseca CF, Oliveira JB, Carvalho RR, Alves LC (2016) Ticks and fleas in crab-eating fox (*Cerdocyon thous*) of Pernambuco state, Brazil. Brazilian Journal of Veterinary Research and Animal Science 53(3): 295–299. https://doi.org/10.11606/issn.1678-4456.bjvras.2016.110634

Torres-Mejía A, de la Fuente J (2006) Risks associated with ectoparasites of wild mammals in the department of Quindío, Colombia. The International Journal of Applied Research in Veterinary Medicine 4(3): 187–192.
